# Solid-Phase Synthesis of Fluorescent Probes for Plasma Membrane Labelling

**DOI:** 10.3390/molecules26020354

**Published:** 2021-01-12

**Authors:** Shuo Zhang, Annamaria Lilienkampf, Mark Bradley

**Affiliations:** EaStCHEM School of Chemistry, University of Edinburgh, Joseph Black Building, Edinburgh EH8 9YL, UK; s1732114@sms.ed.ac.uk (S.Z.); annamaria.lilienkampf@ed.ac.uk (A.L.)

**Keywords:** solid-phase synthesis, cell membrane staining, fluorescent probe

## Abstract

The cellular plasma membrane plays a fundamental role in biological processes, including cell growth, signaling and transport. The labelling of the plasma membrane with targeted fluorescent probes offers a convenient and non-invasive way to image the morphological changes and dynamics of a membrane in real-time and, despite many examples of fluorescent plasma membrane probes, a “universal targeting/anchoring moiety” is still required. In this study, a small number of stearic acid-based probes labelled with 6-carboxyfluorescein was designed and fabricated via solid-phase synthesis in which variations in both charge and hydrophobicity were explored. To ease the synthesis process, a gram-scale synthesis of the Fmoc-Lys(6-carboxyfluoresein diacetate)-OH building block was developed, allowing the discovery of optimal probes that carried a positively charged amino group and a stearic acid tail that exhibited intense plasma membrane brightness and robust retention.

## 1. Introduction

Fluorescent imaging of various cellular systems and components enables the detailed analysis of cellular behaviour and modulation. Amongst these is the plasma membrane, which provides both a natural barrier and a door for communicating with the extracellular environment. Imaging the cell membrane while monitoring its dynamics by fluorescent probes offers a non-invasive, highly sensitive and semi-quantitative mean of exploring and unravelling various cellular processes and cell signaling pathways. A variety of fluorescent probes targeting the plasma membrane exist, with an efficient membrane anchoring moiety typically needed to achieve robust and stable membrane labelling. The cellular membrane can be targeted by probes that interact with a variety of moieties, and this includes membrane glycoproteins (e.g., wheat germ agglutinin-based lectin probes) [1] or antibody-linked fluorophores that recognise specific cell-surface markers [2]. Probes that interact with the cell surface via hydrophobic and/or electrostatic interactions are commonly used, including the long-chain (C-18) dialkylcarbocyanine dye (DiR, 1,1′-dioctadecyl-3,3,3′,3′-tetramethylindotricarbocyanine iodide) [3], though poor water solubility can limit its application. Cholesterol [4] and phospholipid [5] conjugates are good candidates for the plasma membrane, targeting natural lipids and interacting and entering the membrane phospholipid bilayer via hydrophobic interactions. Additionally, many plasma membrane probes rely on electrostatic interactions that promote prolonged membrane staining, with the choice of charged moiety varying between probes. The outer leaflet of a cell surface is rich in negatively charged groups due to sialic acid-rich glycoproteins and phospholipids [6], and the incorporation of positive-charged moieties can help to drive anchoring efficiency. Thus, the commercial lipophilic membrane dye DiR and fluorescent styryl pyridinium dyes, e.g., FM1-43 (*N*-(3-triethylammoniumpropyl)-4-(4-(dibutylamino) styryl) pyridinium dibromide [7,8]) are all positively charged at physiological pH. Similarly, the conjugation of a quaternary ammonium group onto the hydrophobic dye Nile Red produces an efficient cell membrane stain, which remains attached on/in the cell membrane for over 90 min [9]. 

On the other hand, formally negatively charged moieties can result in a poor internalisation of the probes and, consequently, enhanced membrane labelling perhaps not unexpected, in view of a cell surface rich in negatively charged groups. Indeed, the fluorophore *bis*-sulphonate aryleneethynylene gives an efficient labelling of cell membranes, with the lipophilic fluorene ring embedding into the membrane, while the negatively charged sulfonates are solvent-exposed [10]. Indeed, the phenols of fluorescein are typically capped as cleavable esters to allow cellular entry, e.g., the dye Calcein used in live/dead cell assays is made cell-permeable by esterification.

Zwitterionic groups have shown good performance in anchoring and fixing fluorophores onto the cell membrane, with the suggestion of minimising the probes’ “flip-flop” between the leaflets, and their attachment onto/into the outer interface of the lipid bilayer [7,11,12]. Overall, as has been discussed, many plasma membrane dyes/probes exist, and all have individual advantages/disadvantages and specific applications and spectral ranges. As such, considering the broad chemical space these probes occupy, there is a lack of well-defined structural criteria for the design of membrane labelling probes, beyond the need for a hydrophobic anchor.

**Figure 1:**
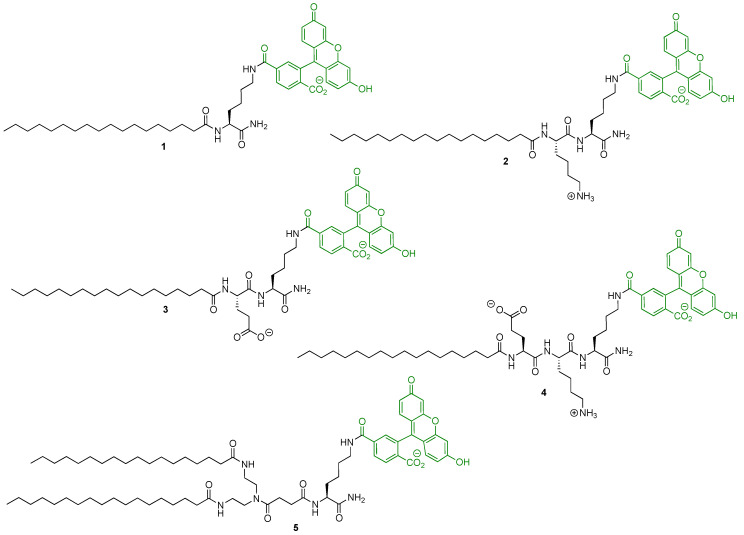
Chemical structures of the fluorescent probes **1**–**5**.

Solid-phase synthesis is an efficient and convenient way to generate compounds, allowing a parallel, step-wise assembly of building blocks (e.g., amino acids) [13]. Herein, we report a solid-phase strategy for the parallel synthesis of fluorescently-labelled probes **1**–**5** (Figure 1), bearing stearic acid chain(s) in combination with charged amino acids, with the compounds evaluated for their plasma membrane labelling efficiency and duration. Stearic acid was pragmatically chosen, being widely found in lipids, inert to peroxidation and readily available and highly affordable. It provided a site of commonality while other changes were made across the compound series. 6-Carboxyfluorescein (λex/em 492/517 nm) was used as the fluorophore due to its biocompatibility, high extinction coefficient (70,000 M^−1^ cm^−1^) and high fluorescence quantum yield (ϕ = 0.85) [14,15] with the building block, Fmoc-Lys(6-Carboxyfluorescein-diacetate)-OH, developed to allow an efficient/clean probe synthesis. Carboxyfluorescein was also chosen, rather than rhodamine-based dyes, as it is negatively charged and as such known not to be cell-permeable.

## 2. Results

### 2.1. Synthesis of Fmoc-Lys(6-carboxyfluoresein diacetate)-OH

**Scheme 1:**
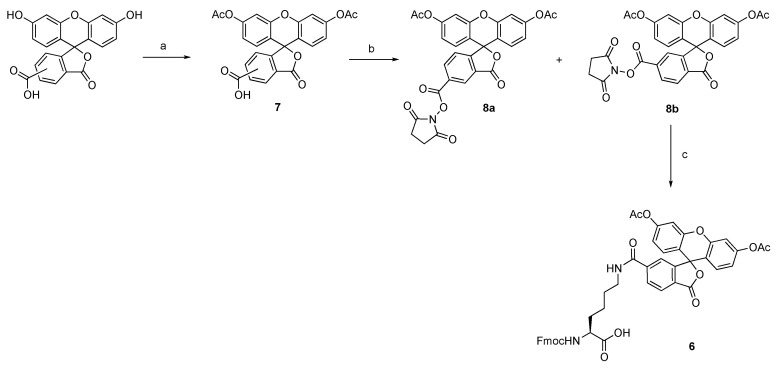
Synthesis of Fmoc-Lys(6-carboxyfluorescein-diacetate)-OH **6**. Reaction condition (a) Ac_2_O, Pyridine, 1 h, 110 °C, yield 95%; (b) DIC/NHS, DCM, 30 min, and column chromatography to separate the isomers (**8a** 28% and **8b** 42%); (c) Fmoc-Lys-OH·HCl, DIPEA, anhydrous DMF; yield 50%.

To allow for the efficient synthesis of fluorescently labelled probes on solid-phase, without the need for orthogonal protecting group strategies, Fmoc-Lys(6-carboxyfluorescein-diacetate)-OH **6** was synthesised on a gram scale (Scheme 1). 5(6)-carboxyfluorescein diacetate **7** was activated as an *N*-hydroxysuccinimide (NHS) ester, with the two regioisomers **8a**/**b** readily separated by column chromatography [16] (**8a** 28% yield and **8b** 48% yield). A single regioisomer (6-Carboxyfluorescein diacetate NHS ester **8b** (2.0 g)) was coupled onto Fmoc-Lys-OH·HCl, in the presence of DIPEA (to quench the HCl salt and NHS generated during the reaction), giving, after column chromatography, Fmoc-L-Lys(6-Carboxyfluorescein-diacetate)-OH **6** in 50% yield. The reaction time (20 min) required a careful control, with extended reaction times resulting in deacetylation of the phenolic hydroxy groups and Fmoc deprotection.

### 2.2. Solid-Phase Synthesis of the Fluorescent Probes

**Scheme 2:**
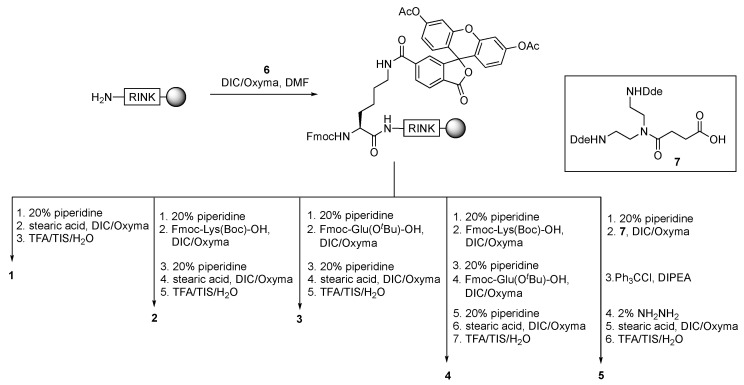
Solid-phase synthesis of **1**–**5**. Coupling conditions for Fmoc-Lys(6-Carboxyfluorescein-diacetate)-OH, Fmoc-Lys(Boc)-OH, Fmoc-Glu(O^t^Bu)-OH: amino acid (3 eq.), DIC (3 eq.), Oxyma (3 eq.) in DMF (0.1 M). Coupling conditions for stearic acid were stearic acid (3 eq.), DIC (3 eq.), Oxyma (3 eq.) in 1:1 DMF/DCM (1 M). Tritylation conditions: Trityl chloride (12 eq.), DIPEA (12 eq.) in anhydrous DCM (1 M) for 12 h × 2. Fmoc deprotection conditions: 20% piperidine for 2 × 10 min. Dde deprotection conditions: 2% hydrazine in DMF for 6 × 10 min. Cleavage conditions: 95% TFA/2.5% TIS/2.5 %H_2_O, shaking for 1 h, precipitation with cold diethyl ether.

The probes **1**–**5** were synthesised on an aminomethyl-functionalised polystyrene resin (0.745 mmol/g, 100–200 mesh), using an Fmoc/^t^Bu strategy (Scheme 2). The resin was functionalised with a Fmoc-Rink linker, and Fmoc-Lys(6-Carboxyfluorescein-diacetate)-OH **6** was coupled using DIC/Oxyma as a coupling combination, to give a resin preloaded with the fluorophore and an Fmoc-protected amino terminus. For the parallel synthesis, this resin (0.8 g) was split into five vessels, followed by the coupling of Fmoc-Lys(Boc) or Fmoc-Glu(O^t^Bu) and finally stearic acid (Scheme 2). For the double-tailed probe **5**, a Dde-protected two-branched amino spacer **7** was synthesised and coupled to the resin [13], before hydrazine mediated Dde-cleavage. The carboxyfluorescein moiety was re-protected on-resin by *O*-tritylation to avoid possible hydrazone formation during the deprotection chemistry [17]. The acetyl-protecting groups of 6-carboxyfluorescein-diacetate were cleaved within 20 min with 20% piperidine, thus not requiring additional deprotection steps during the solid-phase synthesis.

The probes **1**–**5** were cleaved off the resin and deprotected using TFA (with scavengers), and the crude products were precipitated in cold diethyl ether and purified by column chromatography. All the probes (in PBS) had a maximum absorbance between 490 and 510 nm with slightly broadened features and displayed (between pH 5.8 and 7.3) two “peaks”, which we believe is largely associated with anionic/dianionic forms of the dye (see Figure S1). Indeed, a largely single peak was observed at pH 8.0 for probes **1**–**4**, although the two-armed probe **5** displayed a broad peak at all the pH values studied. There is also the possibility that this “broadened shape” is a result (in part) due to probe aggregation, as all the probes displayed different pH-dependent “sizes” (as analysed by dynamic light scattering (see Table S1)). The probes had extinction coefficients ranging between 11,000 and 68,000 M^−1^cm^−1^ and relative quantum yields of 0.08–0.74 (all measured in PBS using fluorescein as a reference dye) (Figures S1 and S2 and Table S1).

### 2.3. Cell Membrane Labelling

**Figure 2:**
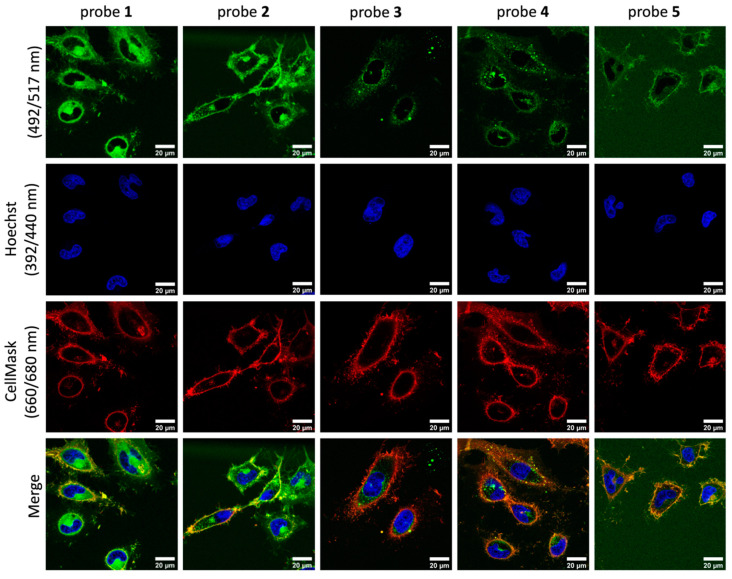
Cell membrane labelling with probes **1**–**5**. Hela cells were incubated with the probes (10 µM) for 15 min and subsequently stained with Hoechst (nuclei stain) and the CellMask Deep Red (commercial dye that stains the cellular plasma membrane), washed with PBS, and the cells were immediately imaged by confocal microscopy. Scale bar = 20 µm. 6-Carboxyfluorescein was used as a negative control and gave little membrane staining or intracellular uptake (data not shown) with identical exposure settings. Filters: Hoechst (λex/em = 392/440 nm), fluorescein (λex/em = 492/517 nm), CellMask (λex/em = 649/666 nm). The fluorescein punctate structure presumably comes from dye accumulation in lysosomes/endosomes or other organelles (but this was outside the scope of our study). The 15-min incubation time was chosen to mimic common membrane staining protocols and for reasons of experimental practicality. For example, CellMask (5–10 min), DiR (1,1′-dioctadecyl-3,3,3,3-tetramethylindotricarbocyanine iodide) (10–20 min).

The cell membrane labelling efficiency of the fluorescent probes **1**–**5** (λex/em 490/530 nm) was evaluated on Hela cells. The cells were incubated with **1**–**5** (10 µM) for 15 min, the cell membrane co-stained with CellMask (λex/em 660/680 nm), and the cells imaged by confocal microscopy (Figure 2). The probes, depending on the overall charge and the number of lipid tails, exhibited different cellular distributions. Probe **2**, with a positively charged lysine (at physiological pH) and a single stearic acid tail, gave the optimal membrane staining (attributed to the electrostatic interactions, as discussed above). Some fluorescence was also observed within the cytoplasm, with a punctuate signal suggesting that **2** was likely accumulating in various organelles. Compared to **2**, probe **3,** with its negatively charged carboxylate group (at pH 7) from glutamic acid, showed weak fluorescence in the cytoplasm, with no cell surface labelling observed. Probe **4**, which incorporated both positively charged lysine and negatively charged glutamic acid side chains, showed a similar cellular distribution to **2** but a weaker overall fluorescence intensity. Probe **1** was distributed throughout the cytoplasm, suggesting an efficient cellular uptake. Comparing probe **1** to probe **5**, two stearic acid lipid tails showed greater membrane selectivity, although with lower fluorescence intensity compared to **2**. With **5**, a much stronger background fluorescence was also observed (after identical PBS washes), most likely due to its poorer water solubility. All the cells were imaged again after 1 h, and probe **2** demonstrated the best membrane staining (Figure S4).

**Figure 3:**
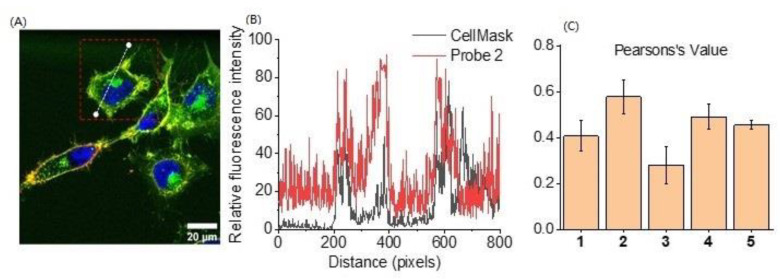
Cellular co-localisation analysis of the fluorescent membrane anchors with the CellMask membrane dye. (**A**–**B**) Example of a plot profile of **2** and CellMask. Relative fluorescence intensities for the green/red channel were recorded along the line drawn across a single cell. (**C**) Pearson’s value of co-localising probes **1**–**5** with the CellMask stain. Data are expressed as mean ± SD; *n* = 5. Image J was used for imaging analysis.

The quantitative co-localisation analysis of the microscopy images showed a well-fitted overlay between probe **2** and CellMask (Figure 3A,B). Using the images Pearson’s values were calculated as a co-localisation coefficient (a perfect co-localisation would give a Pearson’s value of 1, whereas −1 indicates anti-correlation and 0 no correlation). Probe **2** had a Pearson’s value of 0.57 (± 0.07), followed by probe **4** (0.49 ± 0.05), whereas the negatively charged probe **3** had a value of 0.28 (± 0.08). Compared with probe **1** (0.40 ± 0.06), probe **5**, with its double lipid tails, exhibited a slightly higher Pearson’s value (0.4 5 ± 0.02).

**Figure 4:**
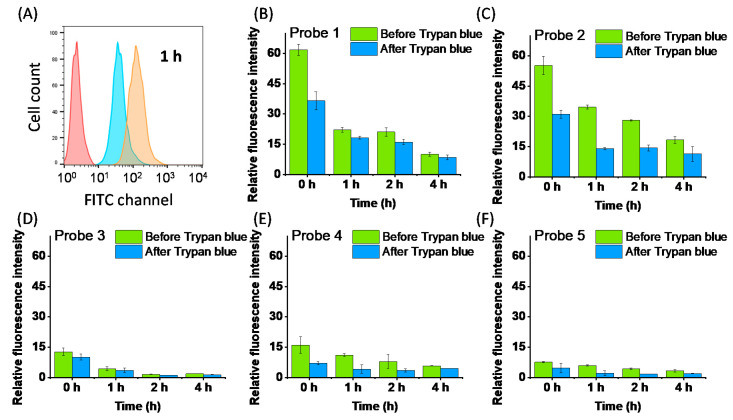
Flow cytometry analysis of fluorescent probes **1**–**5**. Cells were incubated with each probe for 15 min, washed with PBS buffer, incubated in media for different points (0, 1, 2 and 4 h) and detected in the green channel on a Becton Dickinson (BD) FACScan XP5 instrument. After each sample was analysed, trypan blue (0.4%) was added, and the cells were analysed again. (**A**) An example of a flow cytometry histogram of probe **2**. Red cell population: untreated control cells; orange: cells after 1 h of staining; blue: cell after Trypan Blue treatment. (**B**–**F**) Bar graphs representing the relative fluorescence intensity of cells treated with probes **1**–**5** (before and after trypan blue treatment) over the control group (untreated cells) (*n* = 3).

The time-dependent retention of the membrane labelling was evaluated by flow cytometry. After staining with the probes **1**–**5** (15 min at 10 µM), the cells were analysed after 0, 1, 2 and 4 h, with and without Trypan Blue (λ_abs_ 500–600 nm). Trypan Blue has been used exclusively in the literature as a means of distinguishing between surface-bound and internalised fluorescent materials. It functions as a cell-impermeable dye (it is negatively charged) that quenches proximal fluorophores (such as fluorescein), thus allowing the materials attached to the cell surface to be quenched and distinguished from those that have been internalised (non-quenched) [18,19,20,21]. Thus, a high quenching by Trypan blue means high levels of surface immobilisation (and little internalisation), whereas little or no quenching means most of the dye has been internalised into the cells. Directly after staining, all the probes exhibited significantly increased fluorescence labelling compared to cells that were treated with 6-carboxyfluorescein (55 and 16-fold increase in fluorescence for probes **2** and **4**, respectively, which had shown the best membrane labelling via fluorescence microscopy). A clear decrease in fluorescence intensity was seen after trypan blue treatment indicating surface labelling by the probes (Figure 4). Probe **2** showed the highest fluorescence intensity, with clear fluorescence still observed after 2 h (see Table 1).

## 3. Conclusions

Using solid-phase methods, five fluorescent probes were readily synthesised. These were deliberately chosen with varying charges and different numbers of lipid tails, with stearic acid chosen as a common tail denominator to ensure consistency across the series and allowing for functional comparison of the head groups. The solid-phase route was clearly highly-optimal in allowing rapid synthesis, avoidance or minimal purification, and in the future high-throughput library synthesis. Probe 2, bearing a positively charged primary amino group showed the best cell membrane labelling efficiency, with a high fluorescence intensity that was retained over 2 h. Interestingly, the negatively charged (carboxylate) probe resulted in the lowest levels of fluorescence staining, at odds with some previous reports on anionic-based probes, but agreeing with classic observations of poor cellular interactions with negatively charged moieties. An increase in the number of lipid tails resulted in a higher background fluorescence signal compared with the single lipid tail compounds. Overall, these findings begin to offer a framework indicating how best to design plasma membrane targeting fluorescent probes using solid-phase synthesis. This is important, as one of the key issues with many stains now being used across the community is that they are often used without formal knowledge of their structure, as this is often hidden by the manufacturer. Thus, the advantages of our probes are their defined structurex, affordability and robustness and the generation of a structural activity relationship.

## 4. Materials and Methods

*N*,*N*′-diisopropylcarbodiimide (DIC), ethyl cyano(hydroxyimino)acetate (Oxyma), *N*-hydroxysuccinimide (NHS), triisopropylsilane (TIS), 2-acetyldimedone (Dde-OH), 5(6)-carboxyfluorescein, acetic anhydride, stearic acid, trityl chloride, trypan blue (0.4% in PBS), and Dulbecco’s modified Eagle’s medium (DMEM) were all purchased from Sigma Aldrich (Gillingham, UK). Analytical grade solvents were from Alfa Aesar. Aminomethyl Polystyrene Resin, Fmoc-Rink linker and the protected amino acids were purchased from GL Biochem (Shanghai, China) Ltd. Hoechst, CellMask™ Deep Red Plasma Membrane Stain and *N*,*N*-Diisopropylethylamine (DIPEA) were from Fisher Scientific UK Ltd. (Loughborough, United Kingdom). Trifluoroacetic acid was from Fluorochem. Silica gel 60 (0.063–0.200 mm) was from VWR. All compounds were used as received.

^1^H-NMR and ^13^C-NMR spectra were recorded on a Bruker AVA-500 (Bruker, Coventry, UK) (at 500 and 125 MHz, respectively) at 298 K in the solvents indicated (with resonances given in ppm). Low Resolution Mass Spectroscopy (LRMS) was performed on an Agilent LCMS 1100 (Agilent Technologies, Santa Clara, CA, USA) ChemStation with a G1946B quadrupole mass detector, and High Resolution Mass Spectra (HRMS) on a Bruker 3.0 T Apex II spectrometer.

The UV-vis absorption properties of probes **1**–**5** were measured in PBS (pH 7.3) (with 1% DMSO) on a Shimadzu UV-1800 spectrometer (Shimadzu Corporation, Kyoto, Japan). Fluorescence spectra of probes **1**–**5** were measured using a RF-6000 Spectrofluorophotometer. The samples were prepared in the PBS in a fused quartz cuvette (10 mm path), with excitation/emission spectra obtained, measuring excitation/emission bands with a scan speed of 200 nm/min.

Fluorescent cell images were acquired on a Confocal Zeiss LSM880 Airyscan (Carl Zeiss AG, Oberkochen, Germany), and flow cytometry was conducted with a Becton Dickinson (BD) FACScan XP5 (Becton-Dickinson, San Jose, CA, USA). Fluorescent spectra were recorded on a Shimadzu RF-6000 Spectrofluorophotometer.

### 4.1. Synthesis of Fmoc-Lys(6-carboxyfluoresein diacetate)-OH ***6***

6-Carboxyfluorescein diacetate NHS ester **8b** was synthesised according to a published procedure [15]. Fmoc-L-Lys-OH·HCl (2.2 g, 5.4 mmol) and 6-Carboxyfluorescein diacetate NHS ester **8b** (2.0 g, 3.6 mmol) were dissolved in 40 mL anhydrous DMF (0.23 M). DIPEA (1.8 mL, 10.8 mmol) was added dropwise and the reaction mixture was stirred for 20 min. The reaction was quenched with 37 wt.% HCl (1.6 mL, 16.2 mmol), the solution evaporated in vacuo, and the residue purified on a column of silica gel (eluting with 3% MeOH in DCM) to give **6** as a white solid (1.4 g, 50% yield).

^1^H-NMR (500 MHz, CDCl_3_) δ 8.48 (1H, s), 8.24 (1H, m), 7.73 (2H, dd, *J* = 7.6, 2.8 Hz), 7.56 (2H, t, *J* = 8.9 Hz), 7.36 (2H, td, *J* = 7.5, 2.8 Hz), 7.28 (s, 1H), 7.19 (1H, d, *J* = 7.9 Hz), 7.10 (2H, dd, *J* = 3.7, 2.1 Hz), 6.89 (s, 1H), 6.82–6.72 (5H, m), 5.60 (1H, d, *J* = 7.9 Hz), 4.58–4.31 (3H, m), 4.19 (1H, t, *J* = 7.1 Hz), 3.51 (3H, s), 2.31 (6H, s), 1.95 (1H, s), 1.82 (1H, s), 1.71 (2H, m), 1.52 (2H, s). ^13^C-NMR (125 MHz, CDCl_3_) δ 168.9, 168.5, 165.8, 156.4, 154.7, 152.3, 151.7, 144.1, 143.8, 141.3, 137.3, 135.1, 129.1, 127.7, 127.1, 126.6, 125.2, 124.3, 124.1, 120.1, 118.1, 116.1, 110.5, 82.0, 67, 47.2, 39.9, 32.2, 29.8, 28.5, 25.5, 22.5. HRMS (ESI) (*m*/*z*) [M + H]^+^ calcd for C_46_H_39_N_2_O_12_: 811.2498; found 811.2459.

### 4.2. Solid-Phase Synthesis of Fluorescent Probes ***1**–**5***

#### 4.2.1. Synthesis of Lys(6-carboxyfluoresein diacetate)-functionalised Resin

Aminomethyl polystyrene resin (0.8 g, 0.745 mmol/g loading amine, 100–200 mesh) was first treated with Fmoc-Rink-amide linker (0.96 g, 1.8 mmol) using DIC (276 µL, 1.8 mmol) and Oxyma (254 mg, 1.8 mmol) in DMF (6 mL), followed by Fmoc deprotection and the coupling of Fmoc-Lys(6-carboxyfluoresein diacetate)-OH (1.44 g, 1.8 mmol) using DIC (276 µL, 1.8 mmol) and Oxyma (254 mg, 1.8 mmol) in DMF (6 mL). The resin was Fmoc-deprotected (see below) and split into five portions for the synthesis of the fluorescent probes.

#### 4.2.2. General Acid Couplings

The appropriate L-amino acid/stearic acid (3.0 eq. per amine) and Oxyma (3.0 eq.) were dissolved in DMF (1:1 DMF/DCM for the stearic acid coupling), all at 0.1 M, and stirred for 10 min. DIC (3.0 eq) was added and the mixture was stirred for 1 min. The pre-activated mixture was then added to the resin (0.745 mmol/g amine loading,) pre-swollen in DCM, and the reaction was shaken at 35 °C for 1 h. The solution was drained, and the resin washed with DMF (3 × 10 mL), DCM (3 × 10 mL) and MeOH (3 × 10 mL). The completion of the coupling reactions was monitored by a Kaiser test [21].

#### 4.2.3. Fmoc Deprotection

The Fmoc-protected resin was pre-swollen in DCM and 20% piperidine in DMF added and shaken (2 × 10 min). The solution was drained and the resin washed with DMF (3 × 10 mL), DCM (3 × 10 mL) and MeOH (3 × 10 mL) (the Acetyl protecting groups on the fluorescein phenols are removed under these conditions).

#### 4.2.4. Deprotection and Cleavage Off the Resin

The resin (pre-swollen in DCM) was shaken with 95% TFA/2.5% TIS/2.5% H_2_O for 1 h. The resin was filtered and the filtrate precipitated using cold diethyl ether to isolate the product (e.g., compound **5** required 8 h at −20 °C for the precipitate to form).

#### 4.2.5. Purification

Probes **1** and **5** were purified by column chromatography (silica gel, eluting with 10% methanol in DCM). Probes **2**–**4** were further purified by reverse phase chromatography eluting with H_2_O with 10% MeOH to 100% MeOH.

Probe **1**. Stearic acid (96 mg, 0.33 mmol) was coupled to the Lys(6-carboxyfluoresein diacetate)-functionalised resin using DIC (51 µL, 0.33 mmol) and Oxyma (47 mg, 0.33 mmol) in 1:1 DMF/DCM (1.2 mL). Yield: 47%, purify >95% (HPLC). ^1^H-NMR (500 MHz, CD_3_OD) δ 8.43 (1H, s), 8.20 (1H, dd, *J* = 8.0, 1.6 Hz), 7.30 (1H, dd, *J* = 8.0, 0.8 Hz), 6.69 (2H, d, *J* = 2.3 Hz), 6.55 (4H, m), 4.35 (1H, dd, *J* = 9.2, 5.0 Hz), 3.45 (2H, m), 2.24 (2H, m), 1.9–1.2 (18H, m), 0.89 (3H, t) ^13^C-NMR (126 MHz, CD_3_OD) δ 177.2, 176.4, 170.4, 168.3, 161.9, 160.1, 154.4, 138, 135.3, 130.3, 128.7, 125.8, 125.1, 114.0, 111.2, 103.6, 66.90, 54.2, 40.8, 36.9, 33.7, 32.8, 30.7, 30.5, 30.3, 30, 26.9, 26.8, 25.5, 24.4, 24.2, 23.7, 14.4. HRMS (ESI) [M + H]^+^ calcd for C_45_H_60_N_3_O_8_: 770.4375; found 770.4361.

Probe **2**. Fmoc-Lys(Boc)-OH (156 mg, 0.33 mmol) was coupled to the Lys(6-carboxyfluoresein diacetate)-functionalised resin using DIC (51 µL, 0.33 mmol) and Oxyma (47 mg, 0.33 mmol) in DMF (1.2 mL), followed by Fmoc deprotection and the coupling of stearic acid (96 mg, 0.33 mmol), using DIC (51 µL, 0.33 mmol) and Oxyma (47 mg, 0.33 mmol)) in 1:1 DMF/DCM (1.2 mL). Yield: 35%, purify >95% (HPLC). ^1^H-NMR (500 MHz, CD_3_OD) δ 8.50 (1H, s), 8.15 (1H, dd, *J* = 8.0, 1.7 Hz), 7.31 (1H, dd, *J* = 8.0 Hz), 6.76 (2H, d), 6.64 (4H, m), 4.39 (1H, dd, *J* = 9.5, 4.5 Hz), 4.32 (1H, td, *J* = 8.4, 6.1 Hz), 3.47 (2H, m), 2.91 (2H, m), 2.21 (2H, m), 1.9–1.2 (21H, m), 0.89 (3H, *t*, *J* = 6.9 Hz) ^13^C-NMR (125 MHz, CD_3_OD) δ 176.8, 176.4, 174.1, 170.6, 168.4, 161.4, 159.9, 154.1, 138, 135.6, 130.1, 128.6, 125.7, 124.9, 113.7, 110.1, 108.6, 66.9, 54.4, 54.1, 40.8, 40.5, 36.7, 33.1, 32.9, 32.2, 30.7, 30.6, 30.4, 29.8, 28.1, 26.9, 26.7, 25.5, 24.2, 23.7, 23.6, 14.4. HRMS (ESI) [M + H]^+^ calcd for C_51_H_72_N_5_O_9_: 898.5325; found 898.5325.

Probe **3**. Fmoc-Glu(OtBu)-OH (148 mg, 0.33 mmol) was coupled to the Lys(6-carboxyfluoresein diacetate)-functionalised resin using DIC (51 µL, 0.33 mmol) and Oxyma (47 mg, 0.33 mmol) in DMF (1.2 mL). Following Fmoc deprotection, stearic acid (96 mg, 0.33 mmol) was coupled using DIC (51 µL, 0.33 mmol) and Oxyma (47 mg, 0.33 mmol) in 1:1 DMF/DCM. Yield: 35%, purify >95% (HPLC). ^1^H-NMR (500 MHz, CD_3_OD) δ 8.45 (1H, s), 8.21 (1H, dd, *J* = 8.0, 1.6 Hz), 7.29 (1H, dd, *J* = 8.0, 0.8 Hz), 6.70 (2H, d, *J* = 2.3 Hz), 6.57 (4H, m), 4.36 (2H, m), 3.44 (2H, m), 2.41 (2H, t, *J* = 7.5 Hz), 2.22 (2H, m), 2.09 (2H, m), 1.9‒1.2 (20H, m), 0.89 (3H, m) ^13^C-NMR (125 MHz, CD_3_OD) δ 176.8, 176.6, 176.5, 173.9, 170.6, 168.4, 161.5, 159.9, 154.1, 138.0, 135.5, 130.2, 128.6, 125.7, 124.9, 113.7, 110.9, 108.6, 66.9, 54.3, 54.2, 40.9, 36.8, 33.1, 32.8, 30.7, 30.6, 30.5, 30.3, 29.8, 27.9, 26.9, 26.7, 25.5, 24.2, 23.7, 14.4. HRMS (ESI) [M + H]^+^ calcd for C_50_H_67_N_4_O_11_: 899.4801; found 899.4797.

Probe **4**. Fmoc-Lys(Boc)-OH (156 mg, 0.33 mmol) was coupled to the Lys(6-carboxyfluoresein diacetate)-functionalised resin using DIC (51 µL, 0.33 mmol) and Oxyma (47 mg, 0.33 mmol) in DMF (1.2 mL). Following Fmoc deprotection, stearic acid (96 mg, 0.33 mmol) was coupled using DIC (51 µL, 0.33 mmol) and Oxyma (47 mg, 0.33 mmol) in 1:1 DMF/DCM (1.2 mL). Yield: 24%, purify >95% (HPLC). ^1^H-NMR (500 MHz, CD_3_OD) δ 8.45 (1H, s), 8.20 (1H, dd, *J* = 8.0, 1.6 Hz), 7.30 (1H, dd, *J* = 8.0, 0.7 Hz), 6.69 (2H, d, *J* = 2.4 Hz), 6.59 (4H, m), 4.36 (2H, m), 4.26 (1H, dd, *J* = 8.3, 5.8 Hz), 3.46 (2H, m), 2.93 (2H, t, *J* = 7.3 Hz), 2.39 (2H, td, *J* = 7.6, 2.9 Hz), 2.22 (2H, dd, *J* = 14.7, 7.0 Hz), 1.9–1.2 (22H, m), 0.89 (3H, m) ^13^C-NMR (125 MHz, CD_3_OD) δ 176.8, 176.6, 176.4, 174.2, 173.8, 170.7, 168.4, 161.4, 159.9, 154.1, 137.9, 135.6, 130.1, 128.6, 125.7, 124.8, 113.7, 110.8, 103.3, 54.5, 54.4, 54.3, 40.8, 40.5, 36.8, 33.1, 32.8, 32.1, 31.2, 30.7, 30.6, 30.5, 30.4, 29.9, 27.9, 26.8, 26.7, 25.5, 24.3, 23.7, 23.5, 14.4. HRMS (ESI) [M + H]^+^ calcd for C_56_H_79_N_6_O_12_: 1027.5750; found 1027.5727.

Probe **5**. The Dde-protected dual-branched spacer **3** [13] (178 mg, 0.33 mmol) was coupled to the Lys(6-carboxyfluoresein diacetate)-functionalised resin using DIC (51 µL, 0.33 mmol) and Oxyma (47 mg, 0.33 mmol) in DMF (1.2 mL). The resin was washed with 20% piperidine in DMF for 20 min to remove the acetyl protecting groups. The phenolic hydroxyls were reprotected by treating the resin with trityl chloride (12 eq., 410 mg, 1.32 mmol) and DIPEA (12 eq., 250 µL, 1.32 mmol) in anhydrous DCM (2.8 mL, 1 M) for 12 h (×2) [16]. This reaction was monitored by observing the change in resin colour (changing from orange to yellow and the loss of fluorescence). The Dde groups were cleaved by 2% hydrazine in DMF (2 mL, 6 × 10 min) and the resin washed with DMF (3 × 10 mL), DCM (3 × 10 mL). Stearic acid (192 mg, 0.66 mmol) was coupled using DIC (102 µL, 0.66 mmol) and Oxyma (94 mg, 0.66 mmol) in 1:1 DMF/DCM (1.2 mL). Yield: 30%, purify >95% (HPLC). ^1^H-NMR (500 MHz, CD_3_OD) δ 8.43 (1H, s), 8.21 (1H, dt, *J* = 7.9, 1.9 Hz), 7.30 (1H, d, *J* = 8 Hz), 6.69 (2H, d, *J* = 2.3 Hz), 6.61–6.52 (4H, m), 4.31 (1H, m), 3.42 (9H, m), 2.63 (5H, m), 2.13 (4H, m), 1.96 (1H, m), 1.9–1.2 (52H, m), 0.89 (6H, t, *J* = 7.0 Hz) δ ^13^C-NMR (125 MHz, 3:1 CD_3_OD:DCM) δ 177.0, 176.3, 176.2, 174.9, 174.4, 170.5, 167.9, 154.1, 139.9, 137.5, 134.8, 129.9, 125.8, 125.0, 124.0, 117.8, 114.4, 110.9, 103.5, 64.3, 60.4, 57.4, 55.4, 53.5, 49.8, 46.7, 40.6, 38.5, 38.1, 37.0, 36.9, 34.6, 32.8, 31.9, 31.5, 30.5, 30.4, 29.9, 29.6, 29.2, 26.6, 24.0, 23.5, 22.9, 14.3. HRMS (ESI) (*m*/*z*) [M + H]^+^ calcd for C_71_H_109_N_6_O_11_: 1221.8149; found 1221.8149.

### 4.3. Optical Properties of the Fluorescent Probes

The quantum yields were determined using a comparative method with fluorescein as a reference dye (with a ϕ of 0.85 in pH 7.3 PBS) [22]. The quantum yields were calculated following equation Equation (1), where ϕ represents the quantum yield, Grad is the gradient from the plot of integrated fluorescence intensity versus absorbance, and η is the refractive index of the solvent. The excitation wavelength was set at 480 nm, and the emission spectrum was recorded from 490 to 750 nm. The integration of fluorescence intensity was calculated using Origin.

(1) $$\phi_{\mathrm{test}}=\;\phi_{\mathrm{ref}}\;\frac{\left({\mathrm{Grad}}_{\mathrm{test}}\right)}{\left({\mathrm{Grad}}_{\mathrm{ref}}\right)}\frac{\left(\eta_{\mathrm{test}}^{2}\right)}{\left(\eta_{\mathrm{ref}}^{2}\right)}$$

### 4.4. Cell Membrane Labelling of the Fluorescent Probes

Hela cells were grown in DMEM supplemented with 4 mM glutamine, 10% FBS and 100 units/mL penicillin/streptomycin in a 24-well plate. The fluorescent probes **1**–**5** were added separately to a final concertation of 10 µM in media (diluted from a 3 mM DMSO stock solution) and incubated with cells for 15 min. After washing with PBS three times, the cells were stained with CellMask™ Deep Red Plasma Membrane Stain (1:1000 dilution of 5 mg/mL stock solution) and Hoechst (1:1000 dilution of 10 mg/mL stock solution) for 8 min, following the manufacturer’s instructions. The cells were imaged using the Zeiss confocal microscope (λex/λem: Hoechst 392/440 nm, fluorescein 492/517 nm, CellMask 660/680 nm). Pearson’s values of co-localisation of dual-colour fluorescein/CellMask were measured by selecting five single cells in each image and analysed using the *Coloc 2* function to give an average value.

For flow cytometry analysis, the cells were seeded on 24-well plates at a density of 50,000 cells/well in 1 mL of culture media, grown for 1 day, incubated with the fluorescent probes (10 µM) for 15 min, washed three times with PBS and left for 0 h, 1 h, 2 h and 4 h in fresh media. The cells were detached with trypsin/EDTA, harvested in media and analysed. To verify the cellular location of fluorescent probes, 0.4% trypan blue (0.4% in PBS) was added (150 µL per mL of media) into the harvested cell suspensions, shaken for 15 s and analysed. FlowJo was used for data analysis.

## Figures and Tables

**Table 1:** Summary of the five probes synthesised and their cellular labelling profiles.

Probe	+NH_3_	COO−	Stearic Acid Tail	Cellular Labelling Profile
1			single	Strong fluorescence staining with weak membrane selectivity
2	✓		single	Strong fluorescence staining and membrane binding for over 2 h
3		✓	single	Lowest fluorescence staining with no membrane binding selectivity
4	✓	✓	single	Low fluorescence staining compared with Probe 2, with good membrane binding for over 2 h
5			double	Low fluorescence staining and high background signal

## Data Availability

The data presented in this study are available in supplementary material.

## Supplementary Materials

The following are available online, Figure S1. UV-vis of Probes **1**–**5** in PBS (with 1% DMSO). Figure S2. Fluorescence spectrum of Probes **1**–**5** in PBS. Figure S3: co-localisation of probes **1**–**5** and CellMask probe labelling using Plot Profile analysis. Figure S4: Cell membrane labelling of probes **1**–**5** after 1 h. Table S1: Optical properties of probes **1**–**5**.

## Sample Availability

Samples of the compounds are not available from the authors.
