# Supplementary material for: Solid-Phase Synthesis of Fluorescent Probes for Plasma Membrane Labelling
Source: Molecules. 2021 Jan 12;26(2):354. doi: 10.3390/molecules26020354 (PMC7827822; doi:10.3390/molecules26020354)
Supplement: Supplementary file 1 [file molecules-26-00354-s001.pdf]

Supplementary materials

Solid-phase synthesis of fluorescent probes for plasma membrane labelling

Shuo Zhang [1], Annamaria Lilienkampf [1], Mark Bradley [1,*]

[1] EaStCHEM School of Chemistry, Joseph Black Building, University of Edinburgh, Edinburgh, UK;

* Correspondence: mark.bradley@ed.ac.uk

Table of Content

1. Supporting Tables and Figures
2. NMR spectra of compounds **1–6**

Table S1a. Optical properties of probes **1-5**.

Probe	λ_{max}^{Abs} (nm) [a]	ε (M^{-1} cm^{-1}) [a]	λ_{max}^{Ex} (nm) [b]	λ_{max}^{Em} (nm) [b]	φ [b]
1	496	43,140	500	528	0.09
2	499	47,420	498	528	0.17
3	496	62,280	498	526	0.74
4	496	67,900	498	526	0.24
5	494	11,270	496	527	0.08

[a] UV-vis absorption properties and fluorescent of probes **1–5** were measured in PBS containing 1% DMSO (5 μM). [b]Fluorescein was used as a reference (75% (λ_{Ex} = 480 nm, λ_{Em} = 490–750 nm)) [1].

Table S1b. Size distribution of probes **1–5** (5 μM) in PBS at pH's 5.8, 6.9, and 8.0 (buffers wew prepared from 0.1 M Na_2HPO_4 and NaH_2PO_4). Probe solutions were prepared from 0.5 mM DMSO stock solution and 100-fold diluted and size distributions measured on a Malvern Zetasizer.

Size distribution (nm)	pH 5.8	pH 6.9	pH 8.0
Probe 1	955 ± 328	24 ± 6	13 ± 18
Probe 2	1106 ± 163	1106 ± 150	91 ± 7
Probe 3	50 ± 17	122 ± 12	91 ± 0
Probe 4	825 ± 62	190 ± 21	78 ± 8
Probe 5	955 ± 220	295 ± 83	295 ± 109

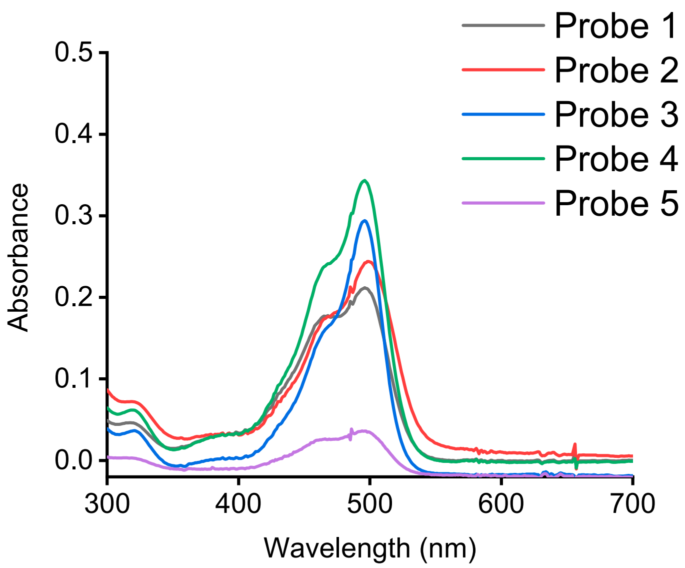

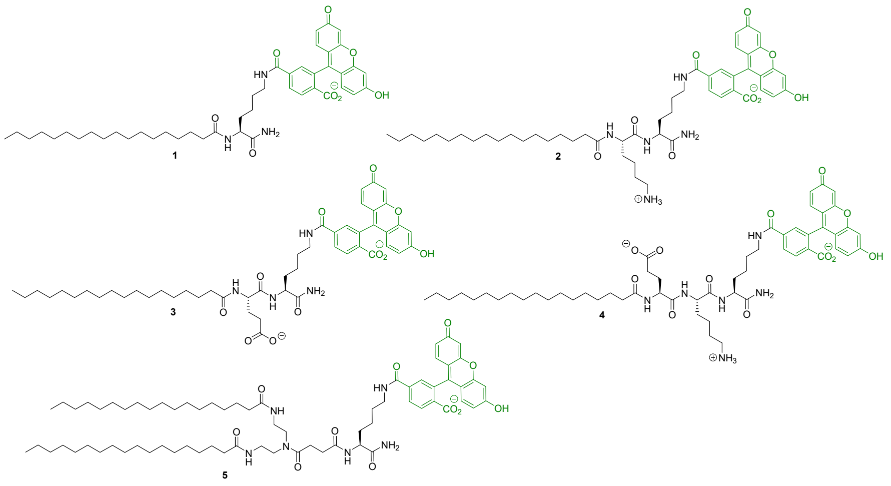

Figure S1a. Absorbance of probes **1–5** in pH 7.3 PBS (5 μM) (prepared by dilution from a 0.5 mM DMSO stock solution). Compound concentrations were determined by ^{1}H NMR intergration using the reference standard 3-(trimethylsilyl)-1-propanesulfonic acid.

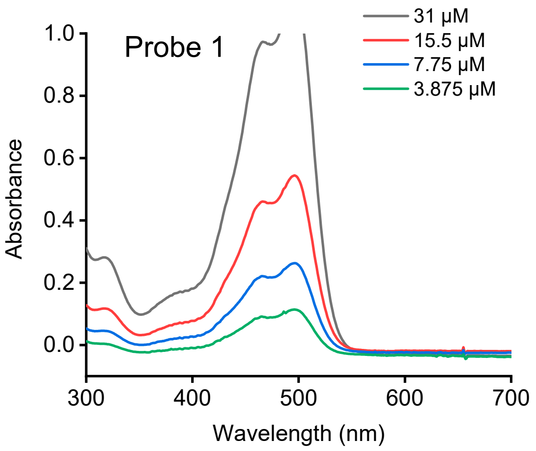
Probe 1
31 μM
15.5 μM
7.75 μM
3.875 μM
Absorbance
1.0
0.8
0.6
0.4
0.2
0.0
300
400
500
600
700
Wavelength (nm)

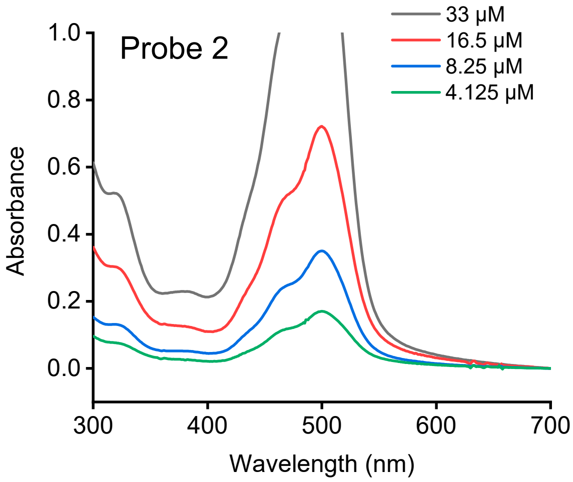
Probe 2
33 μM
16.5 μM
8.25 μM
4.125 μM
Absorbance
1.0
0.8
0.6
0.4
0.2
0.0
300
400
500
600
700
Wavelength (nm)

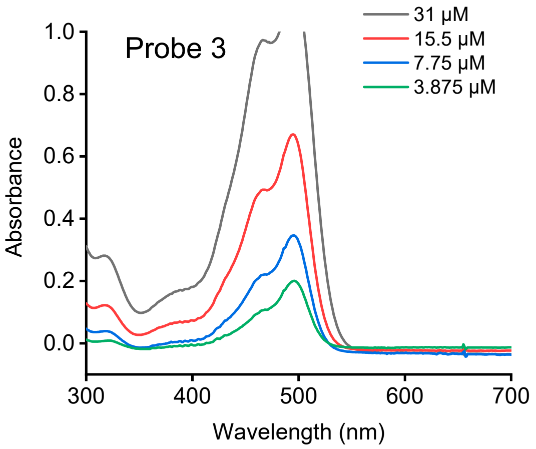
Probe 3
31 μM
15.5 μM
7.75 μM
3.875 μM
Absorbance
1.0
0.8
0.6
0.4
0.2
0.0
300
400
500
600
700
Wavelength (nm)

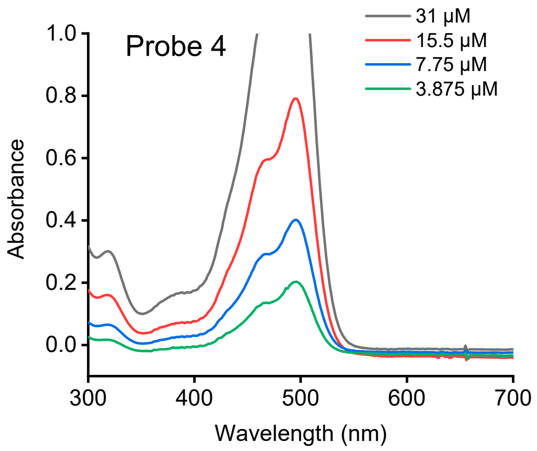

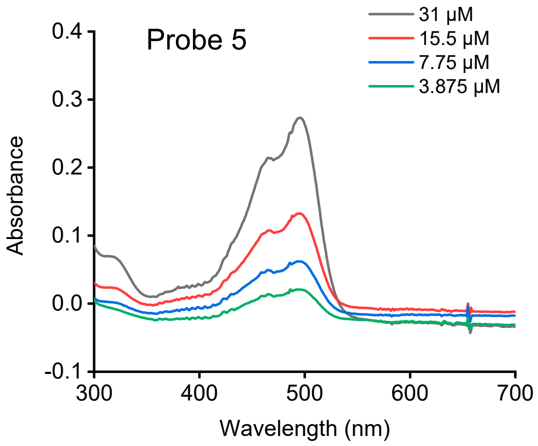

Figure S1b. UV/Vis analysis of the probes **1-5 in** pH 7.3 PBS (5 μM)**.** The fatty acid tail of the probes could allow aggregation in the PBS buffer so dilutions were made and analysed. All probes were prepared from 3.1 mM solutions in DMSO and then 100 fold diluted for the initial measurement and then diluted further three times.

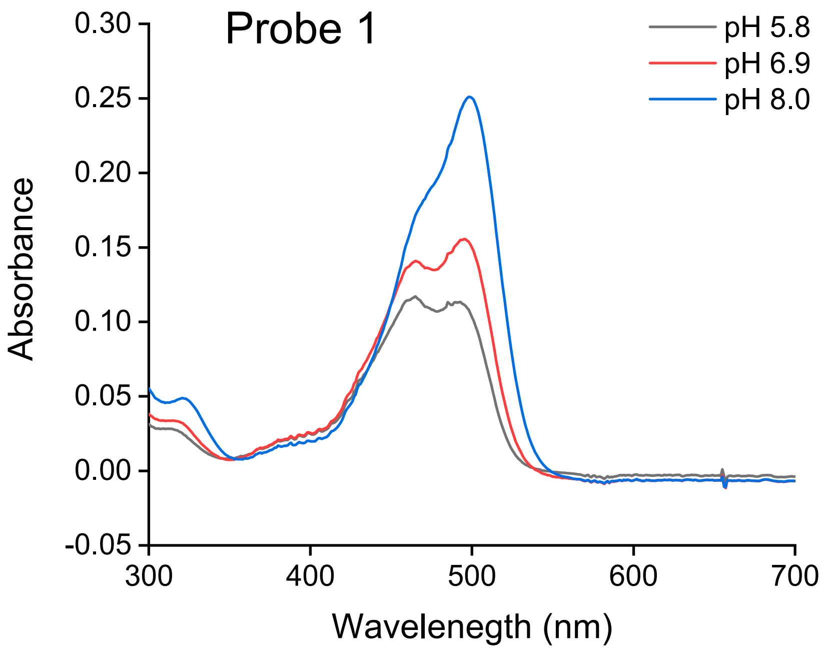
Probe 1
pH 5.8
pH 6.9
pH 8.0
Absorbance
0.30
0.25
0.20
0.15
0.10
0.05
0.00
-0.05
300
400
500
600
700
Wavelenegth (nm)

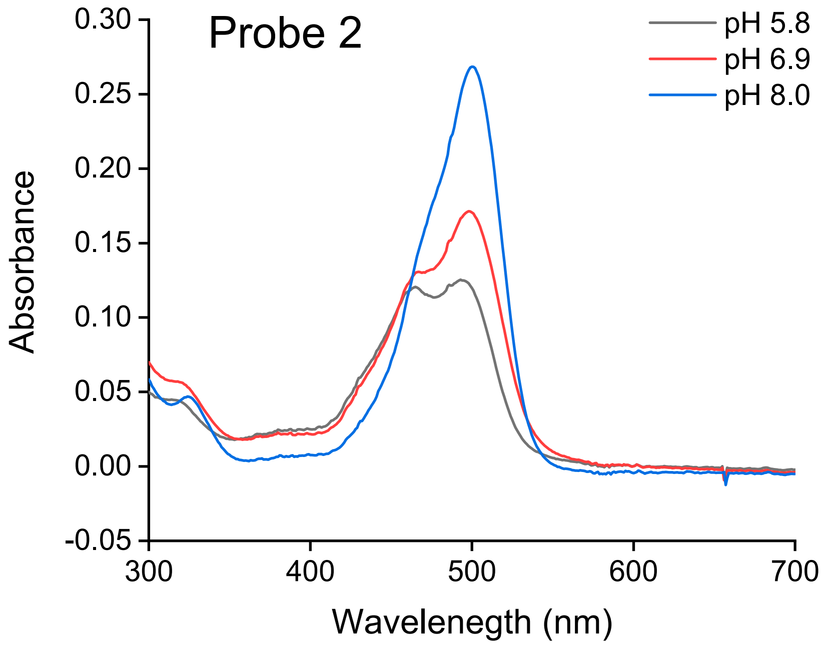
Probe 2
pH 5.8
pH 6.9
pH 8.0
Absorbance
0.30
0.25
0.20
0.15
0.10
0.05
0.00
-0.05
300
400
500
600
700
Wavelenegth (nm)

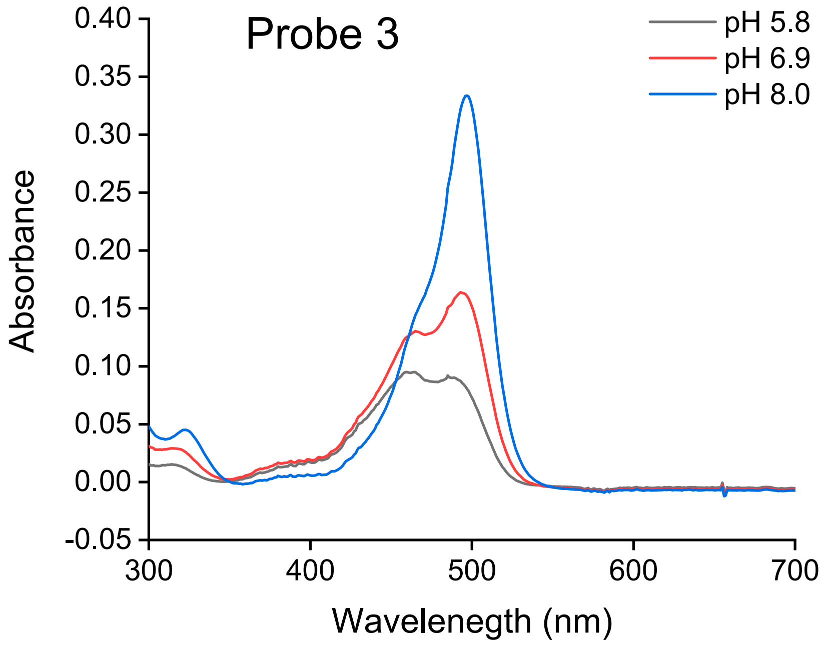
Probe 3
pH 5.8
pH 6.9
pH 8.0
Absorbance
0.40
0.35
0.30
0.25
0.20
0.15
0.10
0.05
0.00
-0.05
300
400
500
600
700
Waveleneгth (nm)

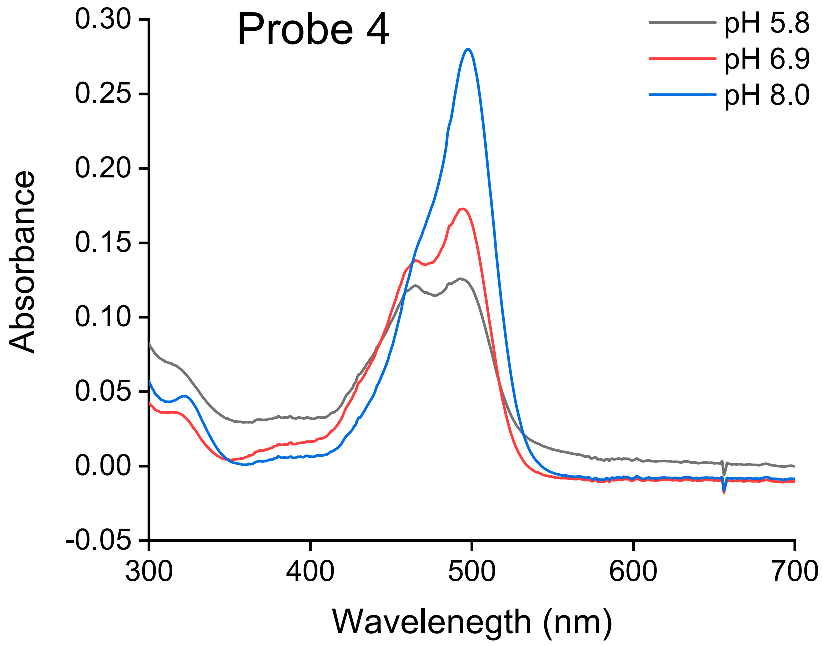
Probe 4
pH 5.8
pH 6.9
pH 8.0
Absorbance
0.30
0.25
0.20
0.15
0.10
0.05
0.00
-0.05
300
400
500
600
700
Wavelenegth (nm)

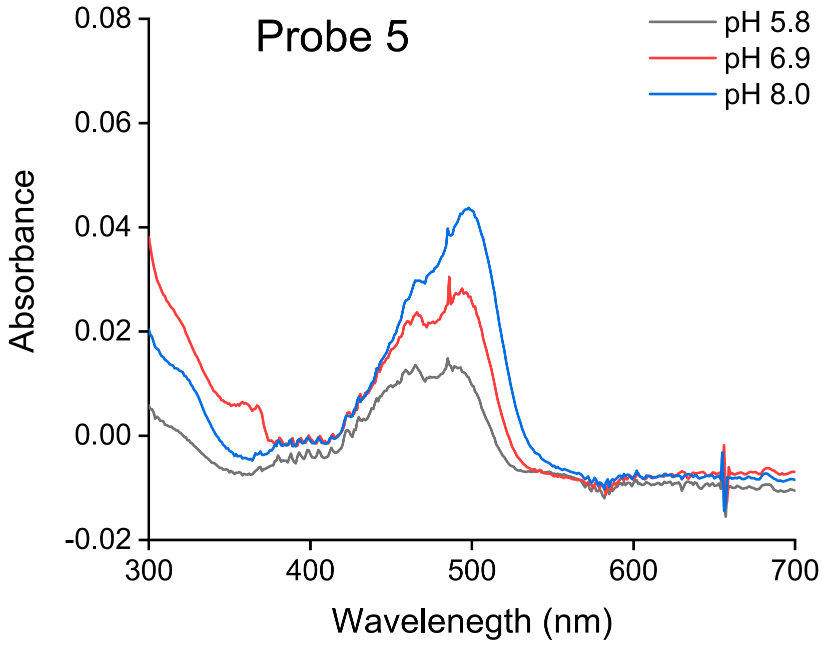

Figure S1c. UV/vis analysis of probes **1–5** (5 μM) in PBS at pHs 5.8, 6.9, and 8.0 (buffers were prepared from 0.1 M Na_2HPO_4 and NaH_2PO_4 solutions). Probes **1–5** were prepared from 0.5 mM DMSO stock solution (and 100 fold dilution into PBS).

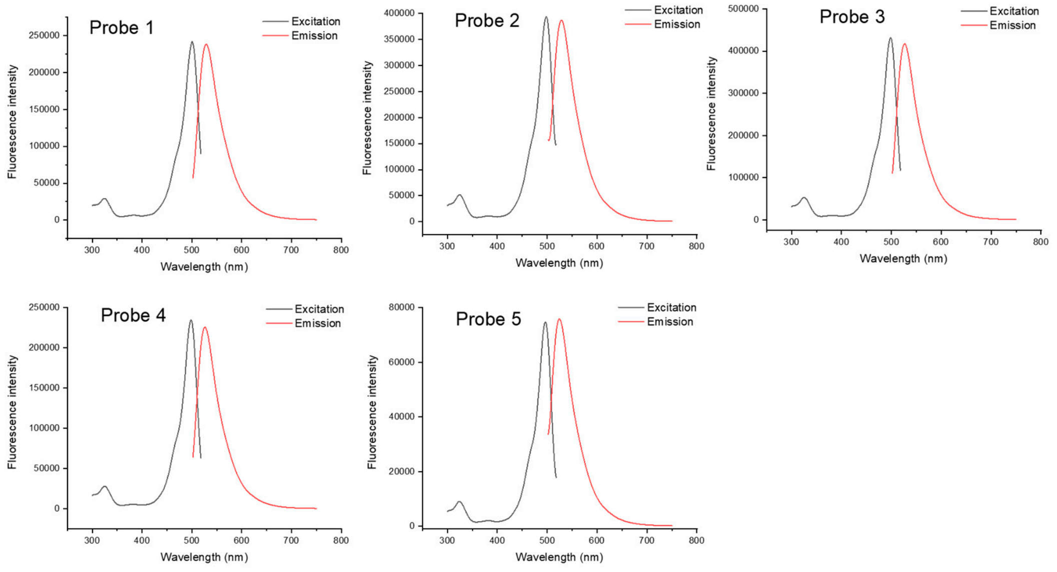

Figure 2. Fluorescence spectrum of probes **1–5**. Probe **1, 2** and **5** (5 μM in PBS, prepared by 100 folds dilution from 0.5 mM DMSO stock solution), probe 3 and 4 (0.5 μM in PBS, prepared by 1,000 folds dilution from 0.5 mM DMSO stock solution).

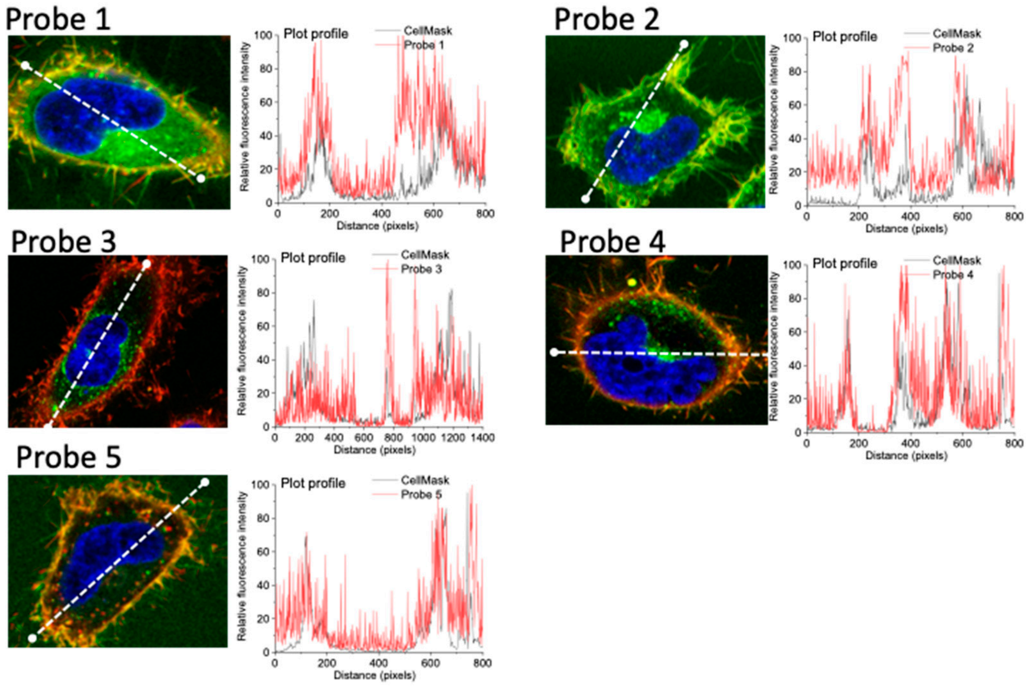

Figure S3. Comparison of probes **1–5** and CellMask labelling using plot profile analysis. A straight line was analysed across a single cell image by confocal microscopy.

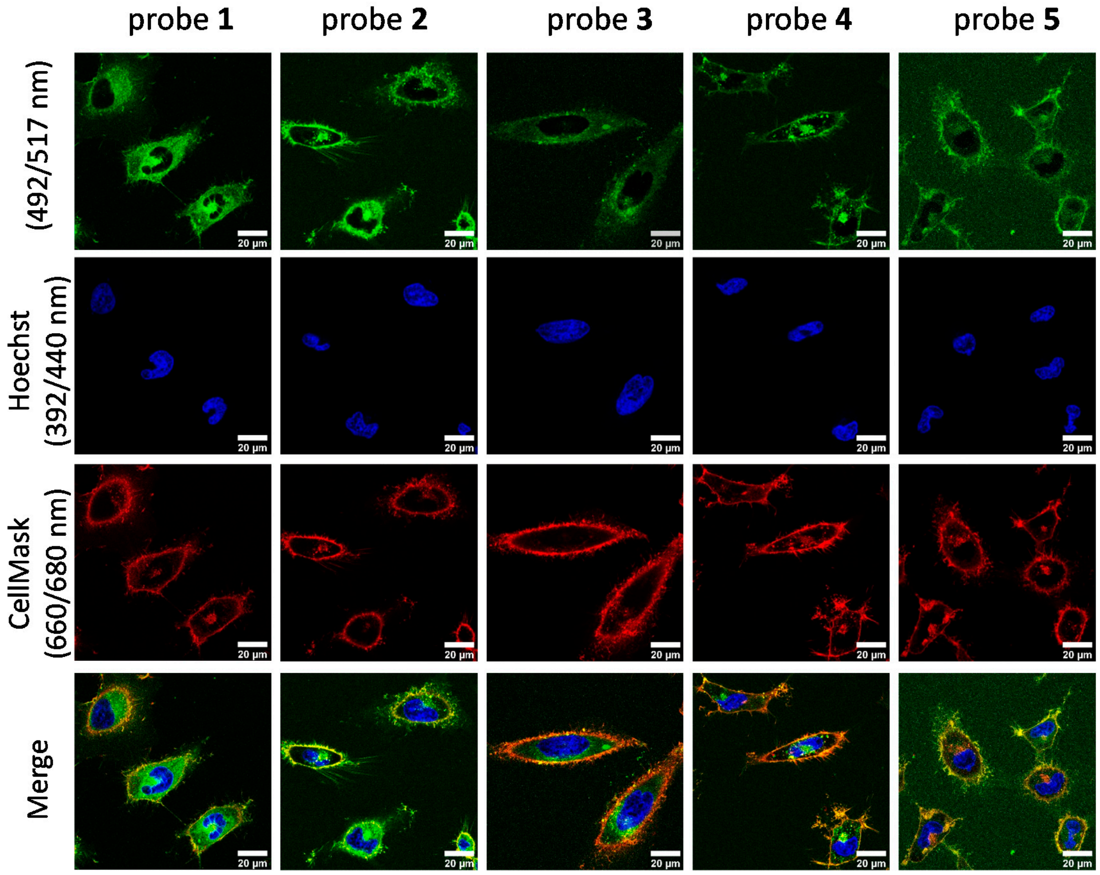

Figure S4. Cell membrane analysis of probes **1–5**. Hela cells were incubated with the probes (10 μM) for 15 min, stained with the Hoechst nuclei stain and the CellMask membrane dye) for 8 min. The cells were incuabted in media for another 1 h and anaysed by confocal microscopy. Scale bar = 20 μm.

NMR spectra of compounds **1–6**

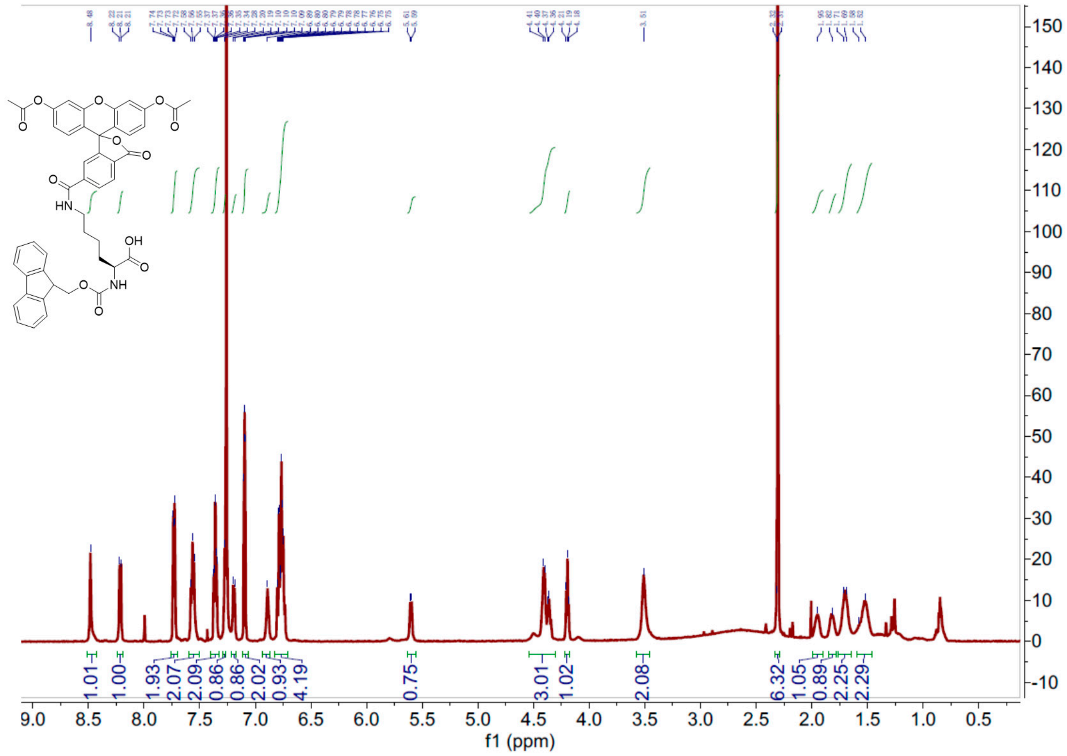

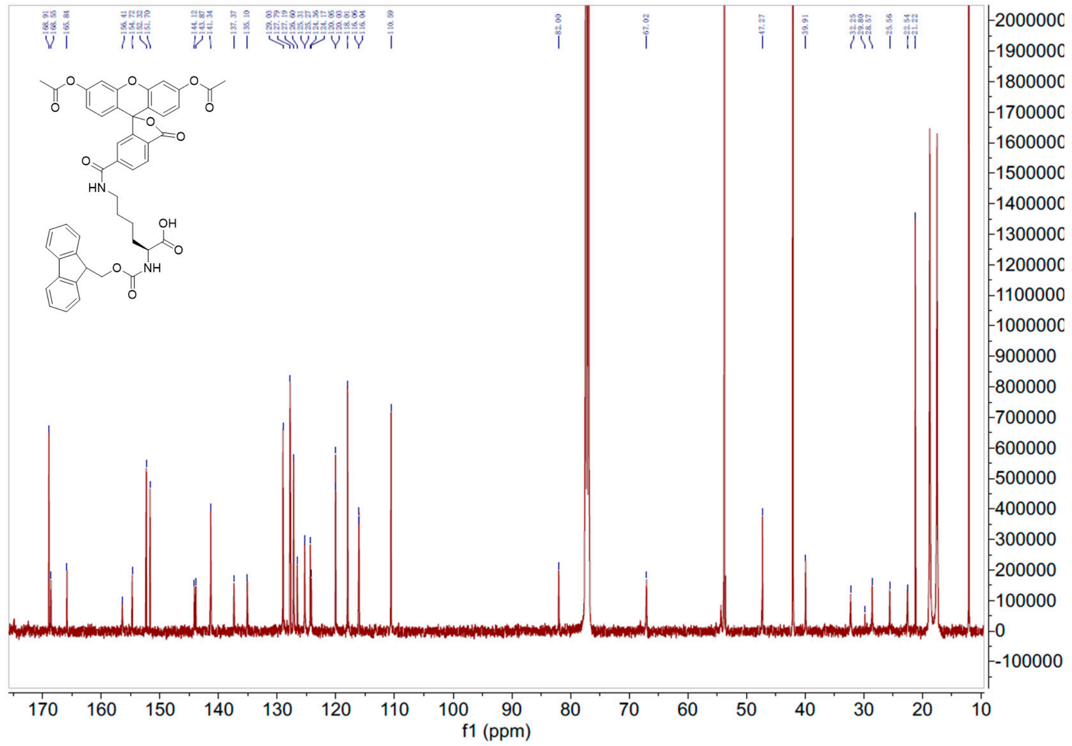

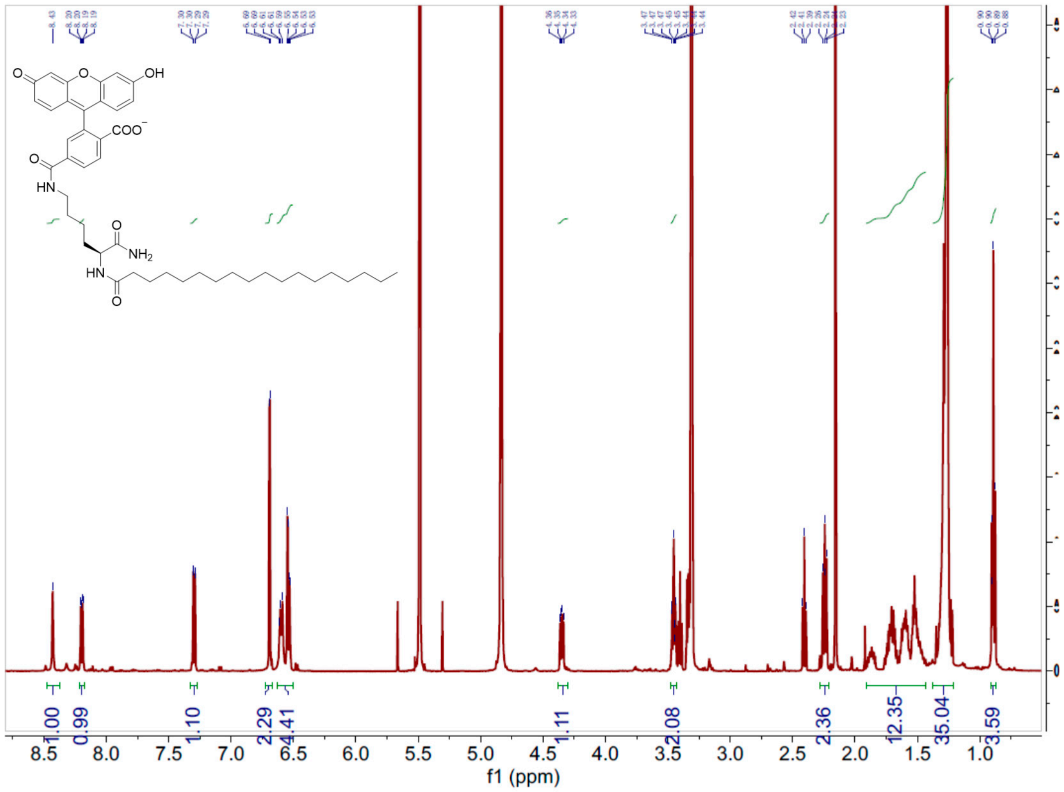

O
OH
COO⁻
HN
NH2
1.00
0.99
1.10
2.29
4.41
1.11
2.08
2.36
12.35
35.04
3.59
8.5 8.0 7.5 7.0 6.5 6.0 5.5 5.0 4.5 4.0 3.5 3.0 2.5 2.0 1.5 1.0
f1 (ppm)

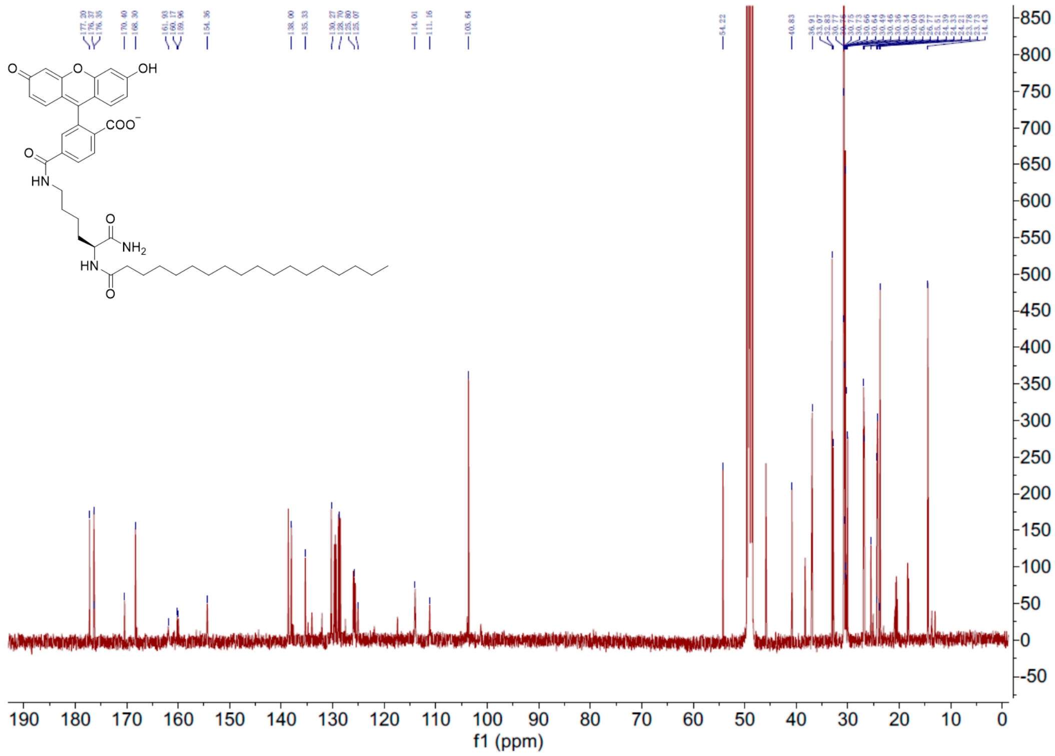

O
OH
COO⁻
HN
NH2
850 800 750 700 650 600 550 500 450 400 350 300 250 200 150 100 50 0 -50
190 180 170 160 150 140 130 120 110 100 90 80 70 60 50 40 30 20 10 0
f1 (ppm)

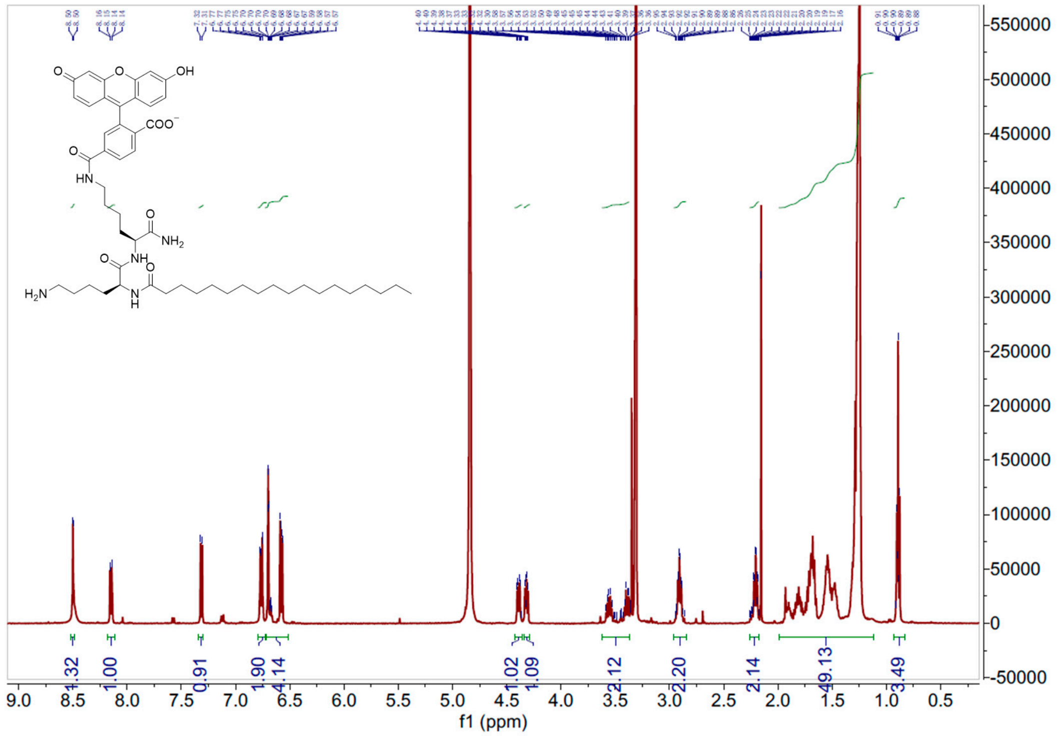

O
OH
COO⁻
O
HN
O
NH2
O
NH
O
H2N
N
H
1.32
1.00
0.91
1.90
4.14
1.02
1.09
2.12
2.20
2.14
49.13
3.49
9.0 8.5 8.0 7.5 7.0 6.5 6.0 5.5 5.0 4.5 4.0 3.5 3.0 2.5 2.0 1.5 1.0 0.5
f1 (ppm)
550000
500000
450000
400000
350000
300000
250000
200000
150000
100000
50000
0
-50000

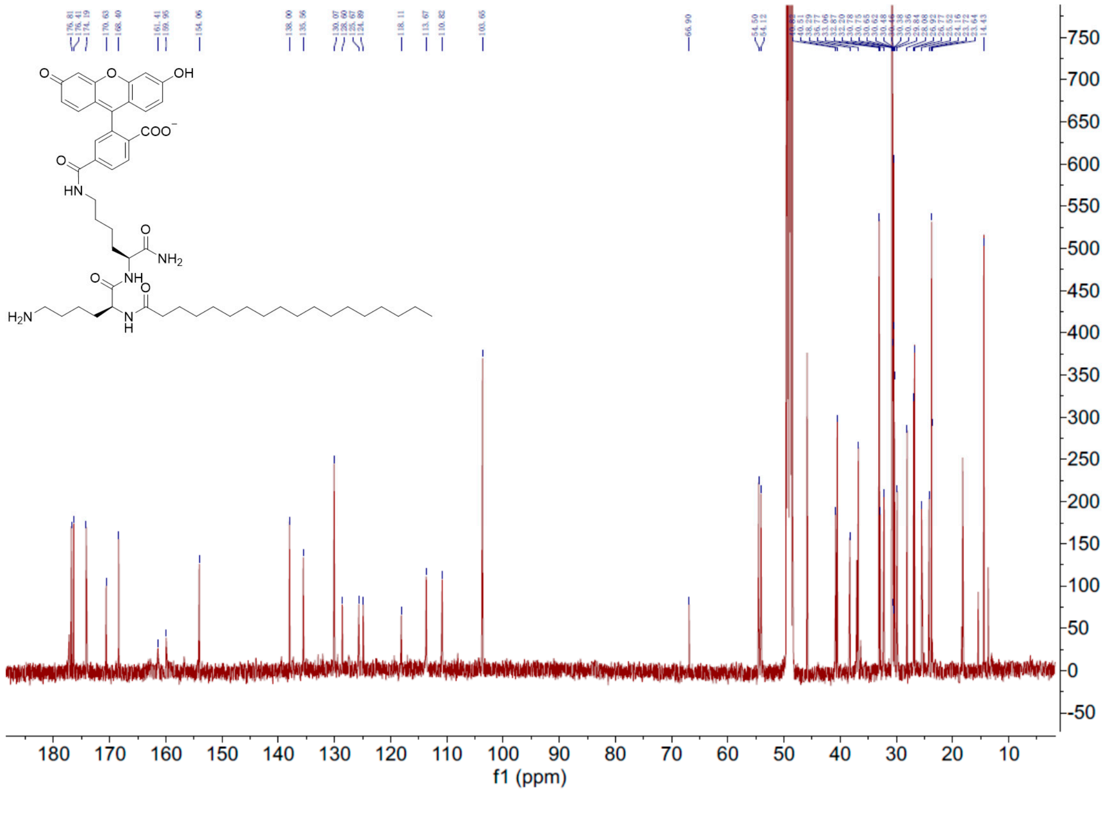

O
OH
COO⁻
O
HN
O
NH2
O
NH
O
H2N
N
H
180 170 160 150 140 130 120 110 100 90 80 70 60 50 40 30 20 10
f1 (ppm)
750
700
650
600
550
500
450
400
350
300
250
200
150
100
50
0
-50

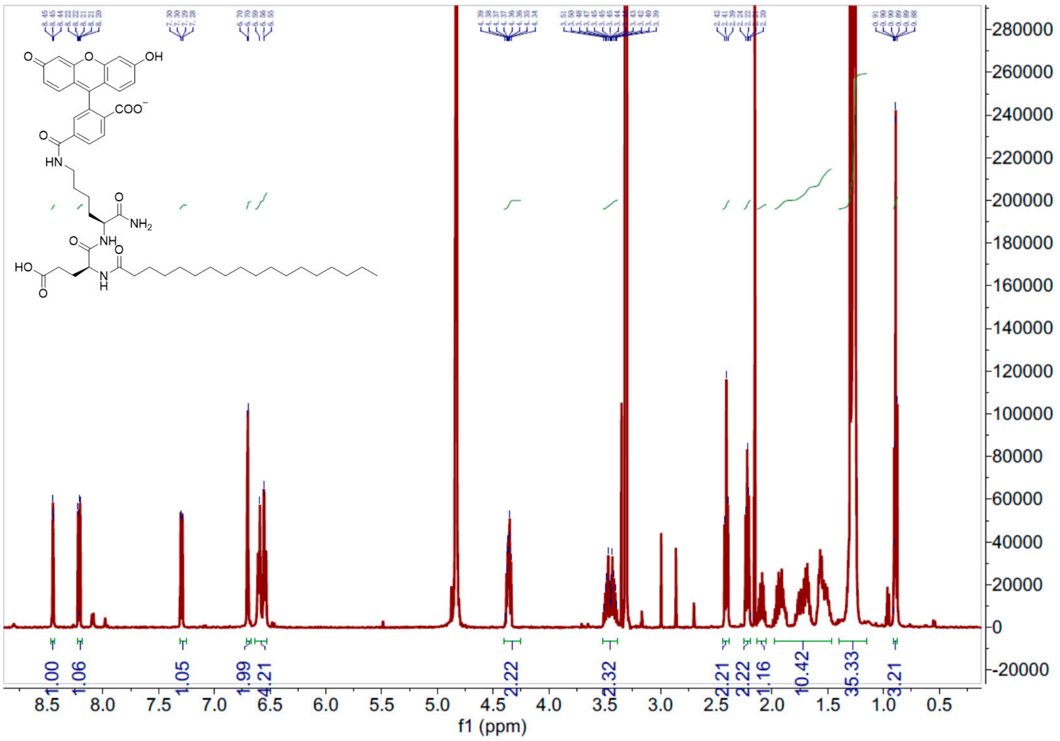
1.00
1.06
1.05
1.99
4.21
2.22
2.32
2.21
2.22
1.16
10.42
35.33
3.21
f1 (ppm)

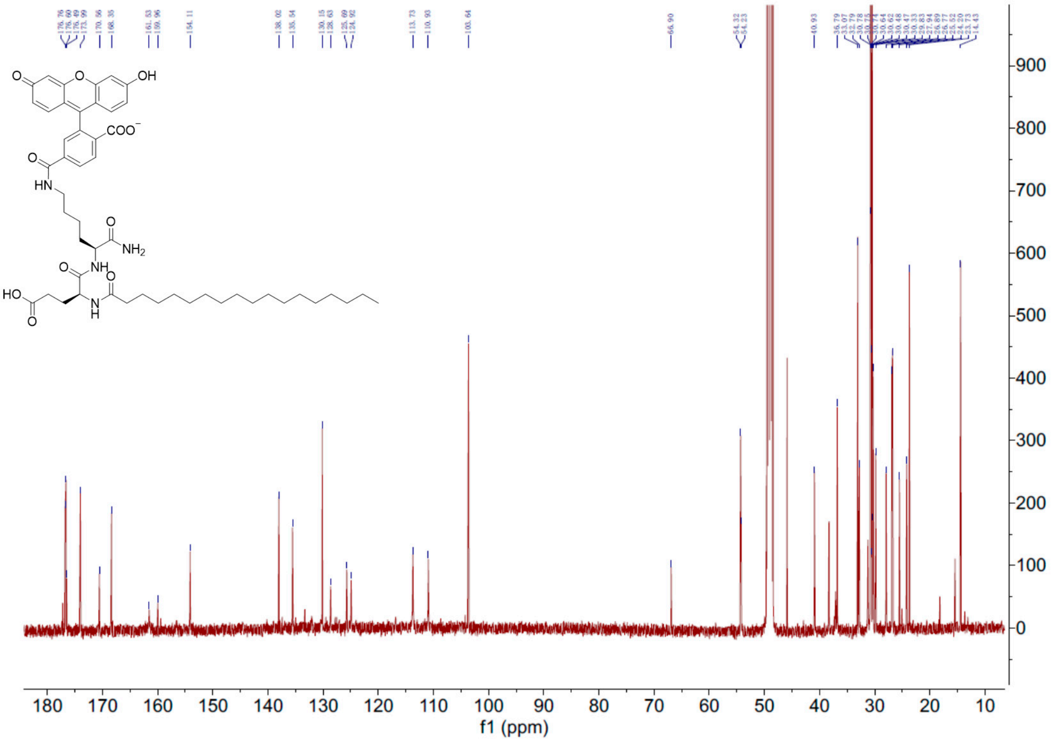
f1 (ppm)

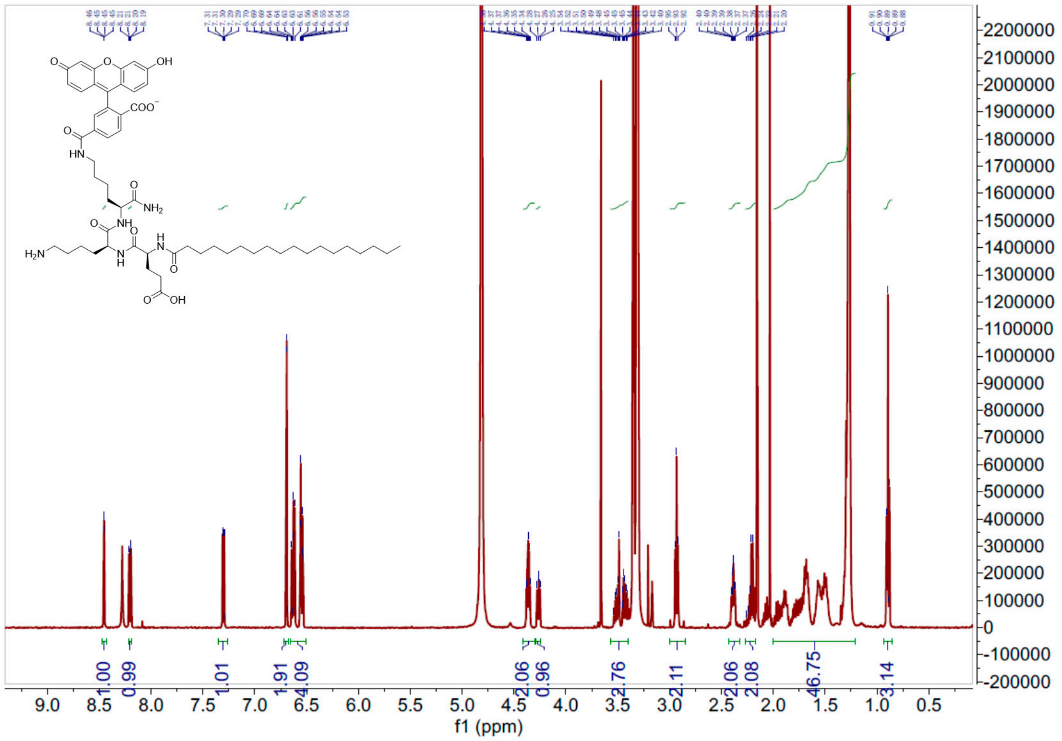

f1 (ppm)

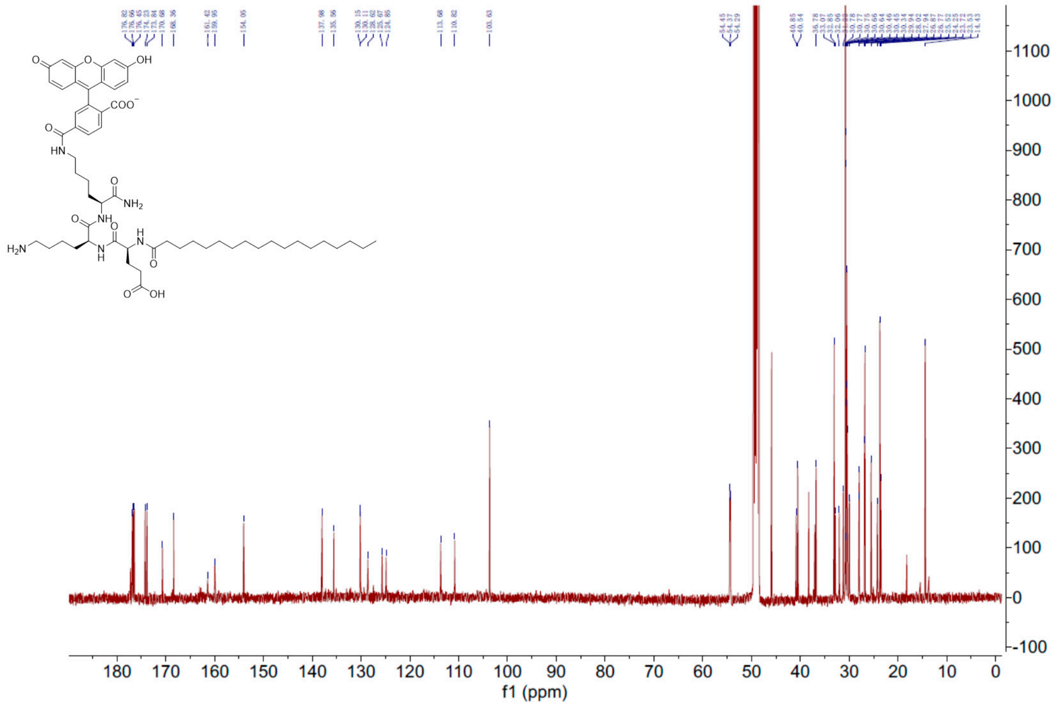

f1 (ppm)

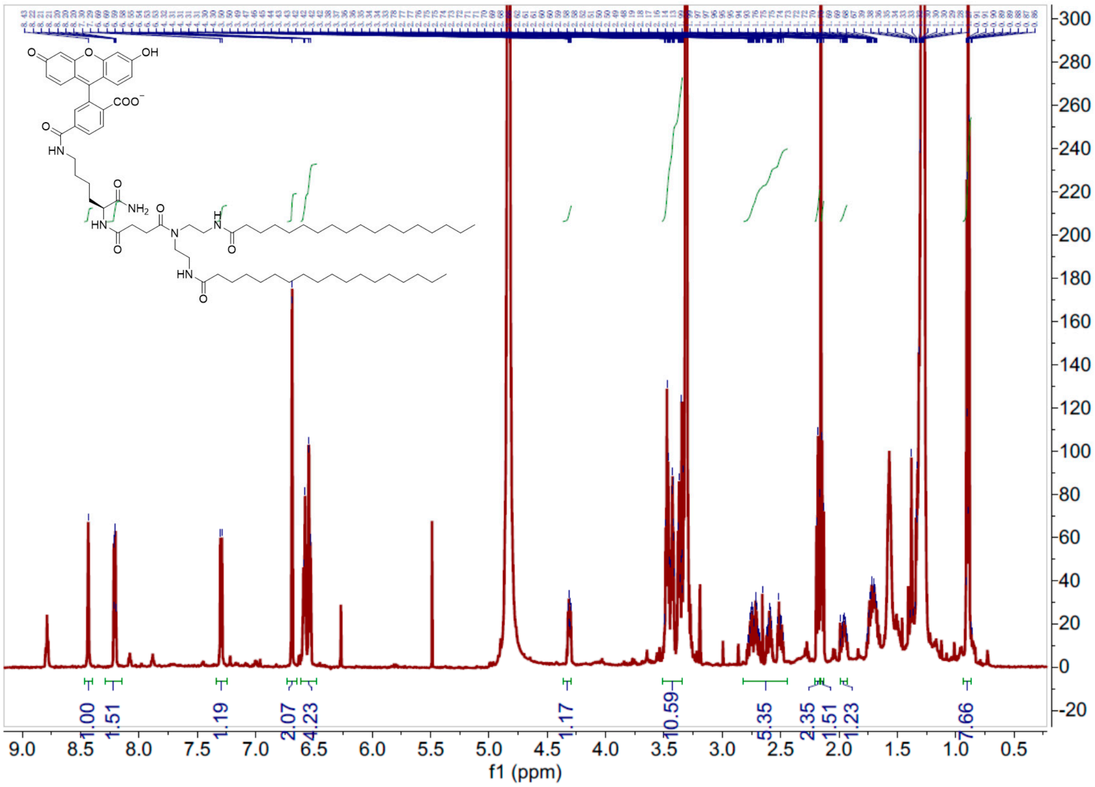

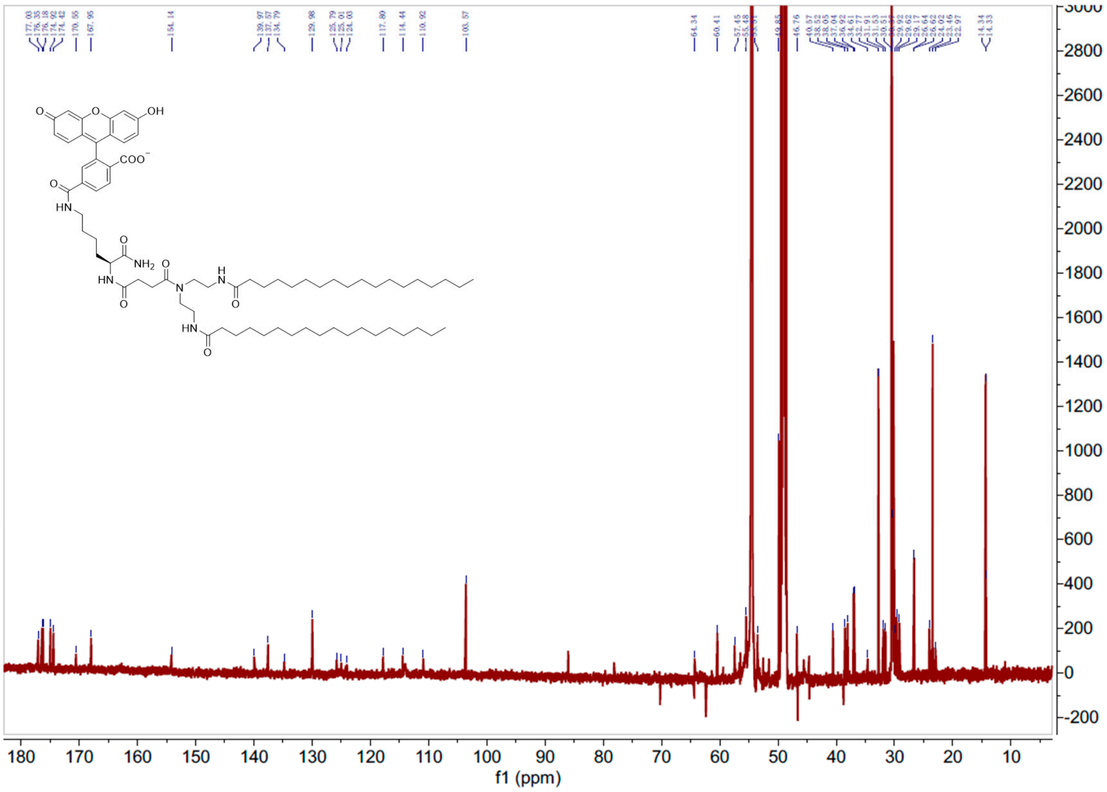

1. Zhang, X.-F.; Zhang, J.; Liu, L. Fluorescence Properties of Twenty Fluorescein Derivatives: Lifetime, Quantum Yield, Absorption and Emission Spectra. Journal of Fluorescence, 2014, 24, 819-826.
